# Editorial: Community series in the role of complement in health and disease, volume II

**DOI:** 10.3389/fimmu.2025.1658763

**Published:** 2025-07-17

**Authors:** Maciej Cedzyński, Thomas Vorup-Jensen, Marcin Okrój, Anna S. Świerzko

**Affiliations:** ^1^ Laboratory of Immunobiology of Infections, Institute of Medical Biology, Polish Academy of Sciences, Łódź, Poland; ^2^ Biophysical Immunology Laboratory, Department of Biomedicine, Aarhus University, Aarhus, Denmark; ^3^ Interdisciplinary Nanoscience Center, Aarhus University, Aarhus, Denmark; ^4^ Department of Cell Biology and Immunology, Intercollegiate Faculty of Biotechnology, University of Gdańsk and Medical University of Gdańsk, Gdańsk, Poland

**Keywords:** complement, classical pathway, alternative pathway, lectin pathway, disease, infection, cancer

The complement system (C’) is a crucial mediator of the innate immune response, interacting with other innate mechanisms protecting the body and with factors of acquired immunity. It also contributes significantly to cell homeostasis, tissue development and repair, reproduction, and interacts with other endogenous cascades, such as the coagulation system. Each of the three major complement activation pathways (classical, CP; alternative, AP; and lectin, LP) employs specific recognition molecules and initiating serine proteases. All of them converge into a common pathway, leading to the formation of the biologically highly active anaphylatoxin C5a and the C5b-9 membrane attack complex (MAC). The latter forms transmembrane channels, either inducing “sub-lytic” activation of the cell or resulting in target cell lysis. Although the complement system was discovered in 1888, it remains the subject of intense investigation, as demonstrated by the present volume. Significant advances are ongoing in various aspects, including its clinical significance and its role in regulating the immune response.

Generally, as an essential branch of first-line defense, complement protects the host from pathogens and abnormal self-derived components. This protection can be achieved through several mechanisms: (i) opsonization by activated factors, (ii) attracting immune cells and enhancing phagocytosis, or (iii) direct lysis after incorporation of the MAC into the cell envelope of the invading pathogen. Therefore, complement plays key roles in (i) preventing the spread of infection to other cells and tissues, (ii) participating in the clearance of damaged cells and tissues, and (iii) preventing the development of chronic inflammation and/or cancer. On the other hand, uncontrolled complement activation may lead to life-threatening effects such as systemic inflammation and shock, dysregulation of coagulation/fibrinolysis, and auto-aggression. Furthermore, under certain conditions, deregulated complement activation may also promote tumor growth and development, also known as tumorigenesis.

The eighteen articles of this Research Topic summarize recent achievements and provide timely reviews in the field of complement research.

## Complement: basic mechanisms, interactions, and cross-talks

Foreign agents that attack the host’s body, including pathogens, their products released naturally or in effect of treatment/immune response as well as broadly understood poisons, both natural (such as animal venoms or plant/fungal toxins) or being the result of intentional human activity, commonly are potent activators of the complement system. Infective microorganisms have developed a variety of evasion strategies, protecting them from C’-dependent opsonization and/or lysis, such as surface-exposed long carbohydrate chains, the release of extracellular vesicles, or binding complement inhibitors, *e.g*. factor H (FH). On the other hand, it is not uncommon that an “evolutionary action” has a corresponding reaction. An example of such a relationship was presented by González-Alsina et al., who demonstrated an elongation factor-Tu-mediated interaction of *Pseudomonas aeruginosa* with factor H-related protein-3 (FHR-3), one of the FH antagonists. As elevated concentration of FHR-3 was associated with higher serum bactericidal activity, it was suggested that it offers protection against *Ps. aeruginosa* infection.

Involvement of complement factors is not necessarily associated with pattern recognition and cascade activation. Bally et al. confirmed (reported elsewhere) the interaction of MASP-2 proenzyme with SARS-CoV-2 nucleocapsid protein. However, no subsequent complement activation was found. On the other hand, incubation of the N protein with active protease resulted in its direct cleavage. The clinical significance of this process remains an open question.

As mentioned, complement system activation may be triggered by toxins or venoms produced by higher organisms. A commonly known (and widely used in research) example is cobra venom factor, a component of the venom of *Naja* species. Although non-toxic itself, it facilitates the penetration of other venom components into the victim’s body, *via* C’ activation. The *in vitro* study performed by Silva de França et al. demonstrated that Africanized honeybee (*Apis mellifera*) venom activates complement in human serum *via* all three canonical pathways (AP predominantly), potently inducing the release of anaphylatoxins and the generation of sC5b-9. This potent stimulation is another example where complement may contribute to life-threatening immunopathological events through envenomation with multiple stings or hypersensitivity. The complement, coagulation, and kallikrein-kinin systems are known to interact to maintain homeostasis and, conversely, contribute to pathology when excessively activated. Their cross-talks reflect the ability of involved proteases to cleave various substrates. Results presented by Lopatko Fagerström et al. indicate that kallikrein-kinin system activation induces C’ activation on primary glomerular endothelial cells *via* bradykinin B1 receptor (B1R) signalling pathway. Data from wild-type and B1/B2 receptor knockout mice demonstrated that the cross-talk is associated with vascular inflammation in the kidney. Collectively, the papers published by Silva de França et al. and Lopatko Fagerström et al. suggest novel possibilities for therapeutic inhibition of the complement system and can serve as an inspiring point for further investigations.

Recently, the complosome (intracellularly expressed components of the complement system) has been gaining growing attention. Using single-cell RNA sequencing, Jarczak et al. demonstrated expression of multiple innate immunity-related genes, including those associated with C’ in human haematopoietic stem/progenitor cells. They suggested that the complosome contributes to the regulation of haematopoiesis *via* the C5a-C5aR-NOD-like receptor protein 3 (NLRP3) inflammasome axis. Moreover, the identified expression of a variety of immune response genes was supposed to be potentially helpful in providing targets for regenerative medicine.

## Complement: clinical associations

The clinical associations of the complement system are complex and multifaceted, ranging from a life-saving multitool to a perpetrator of life-threatening events, from therapeutic agents to therapeutic targets and disease markers. The majority of papers included in this Research Topic have been related to this issue.

### Complement in women’s diseases, pregnancy pathology, and premature newborns

Endometriosis (EM) is a common, chronic, inflammatory gynaecological disorder affecting >10% of women of childbearing age. It is associated with the proliferation of endometrial-like tissue outside the uterus. Apart from such symptoms as dysmenorrhea, pelvic pain, dyspareunia, and dyschezia, it may lead to infertility. Although the etiopathogenesis of EM is still not precisely understood, complement activation is considered to play a role in its development. Agostinis et al. investigated the involvement of C1q in the pathology. The expression of *C1QA, C1QB*, and *C1QC* genes appeared higher in endometrial lesions compared with normal endometrium, which was probably due to the presence of CD68+ cells in the EM microenvironment. C1q was demonstrated to promote angiogenesis in endothelial cells isolated from endometrial ovarian cysts as well as from healthy ovaries and to induce cell motility through gC1qR (receptor for the globular head of C1q). From this perspective, the C1q-gC1qR axis was suggested as a potential therapeutic target in EM. On the other hand, C1q may contribute to the physiological angiogenesis in the ovary.

Dysregulation of the complement system, or its overactivation, for example, as the result of intrauterine infection, is often associated with complications of pregnancy, preterm births, and adverse effects of prematurity. Data summarized and discussed in the review by Balduit et al. confirm the key role of C’ in pre-eclampsia (PE). Based on the literature, the Authors underlined the role of both excessive activation (as evidenced by lower C3 and C4 but higher C4d, C3a, C5a, factor D, Bb, and C5b-9 levels in cases compared with controls) and impaired regulation (lower factor H in PE). Again, the complement system was suggested as a target for treatment.

Preterm newborns (approx. 11% of live births occur prematurely) are at high risk of perinatal complications. Gajek et al. investigated clinical associations of complement-activating collectins in that group. Low concentrations of collectin-10 and collectin-11 (CL-10, CL-11) in cord serum were associated with birthweight <1.5 kg, low Apgar score, and need for prolonged hospitalisation. Low CL-10 was additionally related to the gestational age ≤32 weeks, fetal growth restriction, and need for intensive care >4 days. Decreased concentrations of both CL-10 and CL-11, as well as mannose-binding lectin (MBL), appeared to be risk factors for respiratory distress syndrome (RDS). Similar relationships were found for some *COLEC11* and *MBL2* polymorphisms. The data presented suggest an important role of complement-activating collectins in maintaining homeostasis in preterm neonates.

### Complement in infections

The complement system is considered to constitute the first-line antimicrobial defence branch, responsible for serum microbicidal activity and cooperation with other mechanisms of innate and acquired immunity. In the paper cited above, Gajek et al. demonstrated an association between low CL-10 concentration in cord serum and perinatal early-onset infections.

As mentioned, González-Alsina et al. suggested FHR-3 to protect against *Ps. aeruginosa* systemic infections, as it enhanced the killing of clinical strains isolated from blood. On the other hand, it appeared ineffective against highly serum-resistant bacteria, therefore it cannot be considered “the perfect killer”.

The retrospective analysis performed by Koami et al. revealed that a low CH50 value (<25 U/ml) in patients hospitalised due to infections (CH50 determined within 1 week of admission) was associated with multiple organ failure and coagulopathy, and predicted fatal outcome. The highest mortality rate was noted in patients having both a low CH50 value and C3 concentration. The Authors concluded that early recognition of low complement activity may be useful for risk identification and the prevention of organ failure, thereby improving the outcome.

### Complement in age-related macular degeneration

Associations between the complement system and AMD were investigated by Armento et al. and Omori et al., using retinal pigment epithelium (RPE) cells and mice as models, respectively. In the first-mentioned study, it was found that RPE cells homozygous for the H variant, corresponding to the factor H (*CFH* gene) Y402H polymorphism, are more susceptible to oxidative stress induced by hydroquinone compared with Y-homozygous counterparts. However, normal serum or purified FH had no greater effect on the cell response (Armento et al.). The other study demonstrated that MASP-1-, MASP-3-, and MASP-1/3-null mice exhibited milder symptoms of macular degeneration (RPE depigmentation and destruction, trophy of the photoreceptor layer (PL), and thinning of the outer nuclear layer) after intravenous injection of NaIO_3_, compared with WT animals. Significantly lower C3 activation was observed in MASP-3- and double-knockout mice, suggesting that MASP-3 (and consequently the alternative pathway) is the key player and a candidate therapeutic target in dry AMD (Omori et al.). The role of complement-related gene polymorphisms in AMD pathogenesis was further discussed in a review by Alic et al. In addition to the well-characterized *CFH* Y402H variant, they highlighted several other variants (both risk-associated and protective) of genes encoding complement factors, such as *FB, C2, C3, FI, CFHR1, CFHR3*, and *C9.*

### Complement and innate autoimmunity

The involvement of complement in the pathogenesis of renal diseases has been documented in many investigations. In this Research Topic, Xu et al. focused on membranous nephropathy (MN). They performed differential expression protein (DEP) analysis, based on proteomic data from patients’ urine specimens. It revealed significant correlations between the concentrations of 27 complement-related proteins and the presence of proteinuria. Furthermore, C1s and collectin-12 (CL-12) correlated with tubular atrophy/interstitial fibrosis and monocyte infiltration, while CD59 appeared to be a predictor of disease remission. The urine levels of specific complement components differed between MN patients and healthy controls, as well as between MN patients and those with IgA nephropathy. It seems likely that some of these components may be considered candidate markers of disease progression or remission.

Systemic sclerosis is an autoimmune disease of connective tissue that affects numerous organs, including the skin, lungs, cardiovascular system, kidneys, and alimentary tract. Yin et al. studied the association of C3 in a murine model, using angiotensin II receptor type 1 (AT1R) as an antigen. The C3^-/-^ mice experienced more severe pulmonary inflammation, accompanied by a higher rate of apoptosis, compared with WT animals. The study suggested an anti-apoptotic and rather unexpected anti-inflammatory role of C3 in the lung during the autoimmune response.

### Complement and the complosome in cancer

As mentioned, the complement system may protect against carcinogenesis or, on the other hand, promote it, depending on multiple endogenous and exogenous factors and conditions. That problem, with emphasis on complosome and non-canonical complement pathways (extracellular activity with no direct involvement of canonical pathways), was discussed in a review published by de Freitas Oliveira-Tore et al. Based on available literature, they concluded that various complement components regulate the tumour microenvironment *via* receptor signalling (*e.g*. C3a-C3aR and C5a-C5aR axis) and cross-talk with stromal cells.

Allogeneic haematopoietic stem cell transplantation (allo-HSCT) is widely used in the treatment of haematologic malignancies, such as leukaemias, multiple myeloma, or lymphomas. Fageräng et al. investigated complement functional activity and sC5b-9 levels in patients with acute myeloid leukaemia undergoing allo-HSCT. Data from multiple samples (the first was taken before conditioning chemotherapy, while the last – 4 weeks after transplantation) revealed that the C’ was fully active within the study period in patients who did not experience complications. In turn, marked changes were observed in cases of infections and endotheliopathy. The challenge of post-HSCT blood with bacteria resulted in a hyperinflammatory cytokine response, which appeared to be reducible *via* C3 inhibition. This finding was suggested to have therapeutic potential.

### Complement in cardiovascular and metabolic diseases

The review published by Kong et al. focuses on the multiple disease associations of AP serine protease, factor D, including arrhythmia, aortic aneurysm, hypertension, coronary heart disease, ischemia/reperfusion injury, heart failure, obesity/dyslipidemia, insulin resistance, and diabetic cardiomyopathy. Thoroughly discussed literature data suggest that FD is a prospective marker of cardiovascular and metabolic diseases and/or a therapeutic target. Another review, by Alic et al., concerns the association of the complement system in general with lipid-mediated pathologies. Apart from the AMD as mentioned above, an involvement of C’ in atherosclerosis, metabolic syndrome, and metabolic dysfunction-associated steatotic liver disease was described. Both papers demonstrate the crucial role of the complement system in systemic metabolism.

### Complement and smoking-related diseases

As pointed out above, C’ may be activated by the intentional introduction of certain products to the body. Adverse effects of both active smoking/vaping and second-hand tobacco smoke exposure are a reason for millions of severe disease cases and premature deaths worldwide. The review by Alarabi et al. focuses on the role of C’ activation, which is often an underestimated relationship in morbidity and mortality. An involvement of C’ in smoking-induced respiratory, cardiovascular, inflammatory, mucosal, and macular degeneration diseases was discussed in detail. The Authors also draw attention to the contribution of thromboinflammation to the pathology associated with the aforementioned complement-coagulation interplay. Such smoke ingredients as tobacco glycoprotein (TGP), particulate matter (PM), and heavy metals were considered to induce C’ activation. It was summarized that the interaction between smoking and complement involves a variety of genetic, biochemical, and environmental factors, affecting the risk of disease development and its outcome.

